# Sleep Apnea, Hypertension and the Sympathetic Nervous System in the Adult Population

**DOI:** 10.3390/jcm9020591

**Published:** 2020-02-21

**Authors:** Shreyas Venkataraman, Soumya Vungarala, Naima Covassin, Virend K. Somers

**Affiliations:** Department of Cardiovascular Medicine, Mayo Clinic, Rochester, NY 55901, USA; venkataraman.shreyas@mayo.edu (S.V.); Vungarala.soumya@mayo.edu (S.V.); Covassin.Naima@mayo.edu (N.C.)

**Keywords:** sleep apnea, obstructive sleep apnea, hypertension, neurogenic, resistant hypertension, sympathetic nervous system

## Abstract

Sleep apnea is very common in patients with cardiovascular disease, especially in patients with hypertension. Over the last few decades a number of discoveries have helped support a causal relationship between the two and even resistant hypertension. The role neurogenic mechanisms play has gathered more attention in the recent past due to their immediate bedside utility. Several innovative discoveries in pathogenesis including those exploring the role of baroreflex gain, cardiovascular variability, chemoreceptor reflex activation and the sympathetic nervous system have emerged. In this review, we discuss the epidemiology of sleep apnea and hypertension and the pathogenic mechanisms contributing to neurogenic hypertension. Furthermore, recent management strategies in addition to continuous positive airway pressure (CPAP), such as upper airway stimulation and renal denervation that target these pathogenic mechanisms, are also discussed.

## 1. Introduction

Sleep has far reaching implications for overall health and wellbeing. Sleep disruption, particularly obstructive sleep apnea (OSA), has been implicated in adverse outcomes ranging from decreased daytime alertness and quality to life to increased hospitalization [1]. Patients with sleep apnea are at an increased risk for a broad range of cardiovascular conditions, including high blood pressure, ischemic heart disease, cardiac arrhythmias, heart failure and strokes. [2] Sleep apnea has especially been implicated in increased blood pressure (BP), including a strong association with resistant hypertension. [3,4] This review explores the role neural mechanisms play in the way sleep apnea contributes to the genesis and development of systemic hypertension in adults, and reviews possible treatment strategies. 

## 2. Definitions and Pathophysiology

In adults, an apnea is defined as the absence of inspiratory airflow for ≥10 seconds. Hypopnea on the other hand, is a decrease in airflow, lasting ≥10 seconds, which is associated with an arousal or a drop in arterial oxygen saturation. Sleep disordered breathing (SDB) is characterized by frequent apneas and hypopneas during sleep. Abnormal respiratory events can be central or obstructive and result from an absence or reduction in brainstem neural output to the genioglossus or the thoracic respiratory muscles.

### Obstructive Sleep Apnea (OSA)

OSA should be considered as a neural disease, as the onset of sleep is the event that causes the hypoxemia rather than the effect of lying in the supine position alone. [5] An obstructive apnea is a ≥10 second interruption in respiration associated with continuing respiratory effort. A diagnosis is made when a patient has an apnea–hypopnea index (AHI: Number of apneas and hypopneas per hour of sleep) of >5 with increased daytime sleepiness.

Small airways lead to increased airflow resistance and greater intra-pharyngeal negative pressure during inspiration and in wakefulness. This excites the laryngeal mechanoceptors, which cause increased activity of the pharyngeal dilator muscles, thereby maintaining the airway [5,6]. Sleep causes the reflex to be attenuated, leading to reduced dilator muscle activity, and eventually causing intermittent collapse of the airway. The consequent hypercapnia and hypoxemia stimulate increasing respiratory effort and eventually causes arousal [7]. 

Changes in lung volumes can also impact pharyngeal patency, where lung inflation causes a longitudinal strain on the pharyngeal airway. The caudal strain tends to harden the airway and decreases collapsibility. Thus, transitioning from the upright to the supine position during sleep causes a decrease in lung volume which increases the risk of apnea [8].

Snoring and wake-time sleepiness are the most common manifestations of OSA; although they are relatively sensitive, they lack specificity [9]. Other symptoms and signs of OSA may include restless sleep, periods of silence terminated by loud snoring, nocturia, fatigue, poor concentration, nocturnal angina, and morning headaches. 

## 3. Epidemiology of Sleep Apnea and Hypertension

OSA is the most common sleep-related breathing disorder, estimated to affect approximately 20–30% of males and 10–15% of females, when OSA is characterized as an AHI >5/h of sleep [10]. The prevalence also varies with ethnicity; OSA is more prevalent in African American populations who are younger than 35 years old compared to Caucasians of the same age group, independent of body weight [11].

Both OSA and hypertension are quite prevalent; about 50% of OSA patients are hypertensive. An estimated 30% to 40% of hypertensive patients also have OSA, often undiagnosed [12]. A major step in determining the association between hypertension and OSA was made by studies of the Wisconsin sleep study cohort, which noted a dose-response association between SDB and hypertension that was independent of known confounding factors [13].

A more recent study by Peppard et al. [10] provided descriptive statistics for 1520 study participants. They estimated that among adults aged 30–70 years of age, approximately 13% of men and 6% of women have moderate to severe SDB (AHI ≥ 15). They also showed relative percentage increases from 14% up to 55% depending on age group, sex and SDB severity level, when comparing data from 1988–1994 to those from 2007–2010.

The association between OSA and hypertension has been shown across patient groups including the elderly, and those with a history of stroke [14,15]. In terms of causation, the Wisconsin sleep study cohort first showed a strong dose-response relationship between OSA and hypertension, estimating that patients with mild OSA (AHI of 5–15 events/h of sleep) had two times the risk of incident systemic hypertension compared to those with an absent AHI (adjusted odds ratio 2.89), while subjects with an AHI of >15 events/h of sleep had nearly thrice the risk (adjusted odds ratio of 2.89) [13].

OSA may not only increase the risk of hypertension, but also the risk of its complications. After adjusting for hypertension as a confounder, patients with OSA were nearly at three times the risk of having a stroke. Additionally, a dose-response relationship was seen here too, where patients with mild OSA were nearly two and a half times more likely to have a stroke, while those with severe OSA were at 3.6 times the risk of having a stroke than individuals without OSA [16].

## 4. Mechanisms of Neurogenic Hypertension

### 4.1. Increased Sympathetic Nerve Activity

The groundwork of cardiorespiratory sympathetic physiology was laid by Ludwig et al. in 1871, where they performed concurrent recordings of intrathoracic pressures and arterial BP changes using early kymographs [17]. These experiments introduced fundamental concepts like; (1) the role of ventral medullary neurons and their role in sympathetic tone, and (2) sinus arrhythmia and the role of the vagus nerve. OSA, with repetitive obstructive events during the night, is a testament to how disordered breathing can cause dysregulation of the autonomic cardiovascular system.

### 4.2. Respiration and the Sympathetic System

Haselton and Guyenet showed increased medullary firing frequency at inspiration with the phrenic nerve generating the respiratory burst in the thoracic sympathetic chain [18]. Hypoxia and hypercapnia cause an increase in the sympathetic–respiratory connection with simultaneous activation of abdominal muscles for active expiration [19]. Bursts of increased sympathetic activity occur simultaneously with the peaks of abdominal activity [20].

### 4.3. Sympathetic Nerve Activity during Sleep in Normal Subjects

Micro-neurographic measurements of intraneural recordings of sympathetic burst frequency correlate with plasma norepinephrine levels and have an advantage of providing continuous moment-by-moment measures of sympathetic activity [21]. These findings have provided important insights into the dynamic changes in autonomic activation during and after apneic events. Normal sleep is a time of low physiological strain, especially in the non-rapid eye movement (NREM) phase of sleep (which constitutes nearly 80% of sleep) where sympathetic nervous activity (SNA) decreases and parasympathetic activity increases [2].

In normal subjects, muscle sympathetic nerve activity (MSNA), BP and heart rate are lower while in deep NREM sleep than while awake [22]. This is also associated with a decrease in BP variability. However, arousal stimuli during NREM provoke K complexes along with bursts of sympathetic nerve activity and transient increases in BP. During rapid eye movement (REM) sleep, however, MSNA is similar to the awake state, with abrupt increases in sympathetic drive and BP [22]. 

### 4.4. Sympathetic Nerve Activity in Obstructive Sleep Apnea

OSA is accompanied by high levels of sympathetic drive, even during resting normoxic wakefulness, and is independent of obesity, likely due to a heightened chemoreflex drive even during normoxia [23]. In contrast to normal sleep, in patients with OSA, BP and sympathetic nerve activity do not fall during sleep [24]. Intermittent apneic events with consequent hypoxemia and hypercapnia, in the absence of lung inflation, result in marked increases in sympathetic outflow, with vasoconstriction-induced surges in BP to very high levels, most evident at the end of apnea. The increases in BP and sympathetic activity are attenuated by continuous positive airway pressure (CPAP) mediated amelioration of apneic events. 

#### 4.4.1. Cardiovascular Variability

Cardiovascular variability, in large part determined by autonomic neural mechanisms, appears to be an important marker of cardiovascular risk. Kleiger et al. [25] initially hypothesized that decreased heart rate variability correlates with increased sympathetic or decreased vagal tone. They used Holter monitoring to evaluate 24-h ambulatory electrocardiographic recordings; average RR intervals were analyzed to determine heart rate variability in patients who survived an acute myocardial infarction. Time domain measures of heart rate variability of less than 50 ms correlated with a relative risk of mortality of 5.3 when compared to patients with a heart rate variability of more than 100 ms. Frattola et al. further demonstrated that the cardiovascular consequences of hypertension may depend on the degree of 24-h cardiovascular variability [26].

OSA has been known to be associated with characteristic heart rhythm disturbances [27]. Compared with obese patients, resting awake and otherwise healthy OSA patients have reduced heart rate variability and heightened BP variability, even in the absence of cardiovascular comorbidities. Moreover, the extent of dysfunctional cardiovascular variability is linked to the severity of OSA [23], such that dysfunctional cardiovascular variability predominantly affects patients with moderate to severe OSA. Additionally, RR interval variability is decreased, and BP variability is augmented in patients with severe OSA, even in the absence of potential confounders like age, body mass index (BMI) and elevated BP [28].

Gula et al. [29] hypothesized that the increased SNA in patients with OSA would disrupt frequency domain measures of cardiovascular variability—specifically increasing the low frequency (LF) power and diminishing the high frequency (HF) power. They observed an increased LF/HF ratio in moderate OSA and a normal ratio in normal patients, but surprisingly also in severe OSA. A high LF/HF ratio is thought to indicate sympathetic prevalence and a low ratio vagal dominance. Since breathing frequency is a powerful determinant of the HF power, abnormal breathing patterns that may occur in OSA patients, both during sleep and wakefulness, make frequency domain measures of cardiovascular variability difficult to interpret. Nevertheless, fast heart rates, increased BP variability, impaired RR interval variability, and increased SNA are consistent with an amplified sympathetic cardiovascular drive in sleep apnea.

#### 4.4.2. Chemoreceptor Reflex Activation

Prolonged hypoxemia may produce persistent changes in cellular activity, leading to tissue dysfunction and death. To counteract this, the peripheral chemoreflex, mediated by hypoxic activation of the carotid bodies, sends stimulatory inputs to the 2^nd^ order neurons of the caudal nucleus tractus solitarius (cNTS) [30]. The cNTS neurons act as an integrative center, which then activates the rostral ventrolateral medulla (RVLM), thus leading to increased sympathetic activity in response to hypoxemia [31]. Routine peripheral chemoreflex activation causes bradycardia in conjunction with hypoventilation. De Burgh Daly et al. observed that peripheral chemoreceptors are excited by a reduction in PaO_2_ and a rise in PaCO_2_ and this reflexively increased pulmonary ventilation and the activated vagus nerves slowed the heart rate [32].

In contrast, when apnea occurs centrally or reflexively, carotid chemoreceptor excitation results in an enhanced bradycardia and even cardiac arrest, but paradoxically does not affect respiration. This is called the diving reflex. The respiratory effect of chemoreflex activation depends on the balance that exists between the hyper-ventilatory effect of the reflex itself and the downstream effects of increased ventilation, such as the activation of lung stretch receptors and the central ventilatory inhibition [32]. In summary, reflex chemoreceptor activation induced by apneic asphyxia causes a bradycardic response that can be both neuro- and cardio-protective. 

#### 4.4.3. Chronic intermittent Hypoxia, Chemoreceptor Hypersensitivity and Tonic Chemoreflex Activation

Initial rodent models exposed to chronic intermittent hypoxia (CIH), showed a continued increase in basal arterial pressures that was prevented by denervation of the carotid body [33]. The CIH-induced hypertension was associated with increased baseline sympathetic activity. There was also an increased frequency of discharge of the RVLM pre-sympathetic neurons [34].

Another rodent model sought to examine the sensory role of the carotid body [35] Peng et al. showed that CIH induces functional plasticity in the carotid body, which manifests as sustained activation of afferent discharge that persists for 1 h after termination of hypoxia. Evidence suggests elevated sympathetic nerve activity could be caused by the constant and increased activation of peripheral chemoreceptors. One theory suggests the neuroplasticity of the cNTS, which leads to the modification of cardiorespiratory control of the sympathetic nervous system [36].

Xie et al. [37] assessed the role of chronic hypoxia in humans, where the effect of this stimuli was assessed in nine healthy individuals where they observed that sympathetic nerve burst frequency remained elevated even after withdrawal of the hypoxic stimuli, in contrast to hypercapnia whose withdrawal reduced sympathetic burst frequency. They concluded that both hypercapnia and hypoxia caused sympathetic activation, but that hypoxia induced prolonged sympathetic activation. 

Narkiewicz et al. showed the effects of chemoreflex deactivation on SNA, heart rate and BP in 14 untreated patients with OSA by administering 100% oxygen for 15 min [23]. They observed that OSA patients with chemoreflex deactivation had decreased heart rate, MSNA and BP, but these did not change during administration of room air (no deactivation). These data suggest that tonic chemoreflex activation even during normoxia, plays a role in elevated heart rate sympathetic drive and BP in OSA. 

#### 4.4.4. Baroreflex Impairment

Arterial baroreceptors in the carotid sinus and aortic arch, relaying in the nucleus tractus solitarius, modulate heart rate and peripheral vascular responses to blood pressure fluctuations, whereby baroreflex activation in response to increased BP, causes decreased SNA, thus causing a drop in BP and heart rate [38,39].

Baroreflex function during sleep is state-dependent, and changes with different sleep–wake phase cycles and their adaptive behaviors [40]. It has been proposed that baroreflex sensitivity increases mildly during light NREM sleep and markedly during deep NREM sleep, while in REM, baroreflex gain is not predictable and may be elevated, unaffected or decreased with regard to NREM changes [38].

Heistad et al. showed in dogs that the inhibition of the baroreflex augments the ventilatory response to stimulation of the peripheral chemoreceptors [39]. In studies in humans, Narkiewicz et al. demonstrated that baroreflex activation blunted chemoreflex responses to hypoxemia more markedly than to hypercapnia. Taken together, these findings suggest that in humans with diseases associated with impaired baroreflexes, chemoreflex sensitivity may be heightened [41,42]. Indeed, in both animal models and in humans with mild hypertension, there is evidence of striking increases in chemoreflex drive. 

A clear impairment of baroreflex control in hypertensive patients with OSA was seen most prominently in the NREM phases of sleep [43,44]. In contrast, a study on normotensive OSA patients showed a selective dysfunction of the sympathetic response to baroreflex deactivation but not to baroreceptor activation independent of factors such as hypertension, obesity and age [45]. In patients with chronic intermittent hypoxia, the set point of the carotid baroreflex was seen to reset to facilitate increased mean arterial pressure after only 30 min of intermittent hypoxia. The baroreflex reset causes the stimulus–response curve to move rightward (to higher levels of BP) and upward (for HR and MSNA) [46]. The combination of reduced baroreflex gain and heightened chemoreflex gain may increase sympathetic responses to apnea and increase daytime sympathetic activation, conceivably predisposing to daytime hypertension.

#### 4.4.5. Role of Genetics

The contribution of genetic polymorphisms in mediating the relationship between hypertension and OSA has been studied by several investigators [47,48]. In hypertensive patients with OSA, the role of β1-adrenergic receptor gene polymorphisms was studied by Bengtsson Boström et al. [49]. They observed that hypertension in mild OSA was associated with an Arg389Gly polymorphism of the β1-adrenergic receptor gene. Furthermore, males with hypertension who had the Arg389Arg genotype were observed to have a higher AHI (age adjusted) than carriers of the Arg389Gly/Gly389Gly genotypes. This association was not apparent in women. Polymorphisms of α2 and β2 adrenergic receptor genes were not associated with OSA in hypertensive patients.

## 5. Management

### 5.1. Continuous Positive Airway Pressure (CPAP)

CPAP therapy results in acute reductions in both sympathetic drive and BP during sleep [24]. In terms of long-term effects of CPAP, multiple randomized controlled trials and several meta-analyses have demonstrated that CPAP results in small but significant decreases in BP in OSA patients [50,51,52], of 2.0 to 2.5 mmHg in SBP and 1.5 to 2.0 mmHg in DBP while using 24-h BP monitoring.

CPAP in and of itself may have limited utility as an antihypertensive strategy, since CPAP alone may not decrease BP if the hypertension is unrelated to OSA. Furthermore, if OSA-induced hypertension is chronic, it may cause vascular bed remodeling with an increased BP set point. However, considering a long-term reduction of 2–3 mmHg in SBP is associated with a 4%–8% decrease in mortality [50], the potential clinical benefit of CPAP therapy in OSA-induced hypertensives is not trivial [12].

Additionally, BP improvement with CPAP therapy is highly variable as severity of OSA, baseline BP and CPAP compliance (a threshold of 4 h or more) determine the reduction of BP levels, and these factors would cause some patients to have better antihypertensive benefits than others [1].

Greater reductions are seen in patients with resistant hypertension, where a 4.7 to 7.2 mmHg reduction was seen in systolic blood pressure (SBP) and a 2.9 to 4.9 mmHg reduction seen in diastolic blood pressure (DBP) [53,54]. In resistant hypertension, a linear correlation was noted between the hours of CPAP use and reduction in BP (decreases of 1.9 and 1.0 mmHg in SBP and DBP, respectively, for each added hour of CPAP use) [55] This greater reduction may be due to the contribution of hypoxemia to the overall increase in BP that is blunted with longer CPAP use. 

Current treatment standards place continuous positive airway pressure (CPAP) as the first line of OSA therapy due to the well based evidence of efficacy and safety, and especially the benefits of treatment in sleepy patients. Unfortunately, despite its documented efficacy, adherence remains a problem with CPAP. The APPLES [56] and HomePAP [57] studies showed adherence rates of only 39–50%. Thus, if only CPAP is considered as a treatment option, a large proportion of the patient population will remain untreated; other modalities should therefore be considered for treatment of OSA and potentially for comorbid hypertension. These include hypoglossal nerve stimulation and pharmacologic anti-hypertensive agents. 

### 5.2. Neurogenic Modulation as a Treatment of OSA

#### 5.2.1. Upper-Airway Stimulation for OSA

The onset of apneic asphyxia is usually accompanied by a decreased stimulatory drive in the upper airway muscles [1,58]; patency of the upper airway correlates with genioglossus muscle stimulation [59]. Recent studies suggest that the collapsibility of the airway is a consequence of neuromuscular dysfunction that mimics denervation injury [60]. Other studies in patients with OSA indicate delayed distal latency in hypoglossal nerve conduction in 75% of patients and a low motor amplitude when compared to controls [61].

The genioglossus muscle is the largest airway dilator muscle and causes tongue protrusion and stiffening of the anterior pharyngeal wall, with genioglossus stimulation causing improvement of pharyngeal collapsibility, and increased upper airway diameter and maximal inspiratory flow [62], along with reduction in apneas and hypopneas in patients with OSA [63]. Implantable upper airway stimulation was first explored in a pilot study where eight patients received an implantable hypoglossal nerve stimulator for OSA [62]. A signal of end expiration was sensed by the respiratory pressure sensor, which then activates the hypoglossal nerve stimulator at the start of inspiration. Sleep studies performed at month 1, 3 and 6 post-operatively showed that most participants (seven of eight) had significant reductions in AHI (from 52.0 +/- 20.4 to 22.6 +/- 12.1(*p* < 0.001)). Electrode breakage and sensor malfunction prevented use beyond 6 months, proving to be a challenge of this technique and thus limiting applicability. 

The next generation devices were investigated in two larger studies [64,65]. Eastwood et al. [64] investigated 21 patients with moderate-severe OSA, who had never had surgery and were intolerant of CPAP. These patients had sleep studies performed at 1, 3 and 6 months, and there was significant improvement in AHI, which fell from 43.1+/- 17.5 to 19.5 +/- 16.7, and the Epworth sleepiness score (ESS) which fell from 12.1+/- 4.7 to 8.1 +/- 4.4.

These studies paved the way for the multicenter prospective Stimulation Therapy for Apnea Reduction (STAR) Trial [66]. A total of 126 patients underwent an implantation of the hypoglossal nerve stimulator and were followed for 12 months to assess efficacy and adverse events. Stimulation therapy elicited clinically and statistically significant improvements in both ESS and AHI. Patients with hypertension were excluded from the study. BP measurements were not used as a primary or a secondary outcome in this study.

Walia et al. compared the effects of positive airway pressure and hypoglossal stimulation on BP. [67]. They observed that after four months of hypoglossal stimulation treatment, patients did not have changes in BP (systolic, diastolic or mean arterial pressure). Positive airway pressure showed a greater improvement in diastolic BP (mean difference of change of 3.7 mmHg) and mean arterial pressure (mean difference of change between groups of 2.8 mmHg) when compared with hypoglossal stimulation. 

In conclusion, neural stimulation of the hypoglossal nerve can potentially be used for the treatment of OSA, with attenuation of apneic events and sleepiness, but does not have any effects on BP.

#### 5.2.2. Renal Denervation and Implications for Resistant Hypertension in OSA

Increased sympathetic nerve activity, a characteristic of patients with OSA, plays a major role in the development of resistant hypertension [68]. It has been hypothesized that renal sympathetic nerves modulate sodium homeostasis, with renal nerve activation enhancing sodium and water retention by altering renal blood flow, increasing renin release and thus aldosterone release [69]. Takahashi et al. [70] used mice models exposed to CIH to show that renal denervation ameliorated CIH-induced hypertension and other hyperadrenergic effects. Therapeutic renal sympathetic denervation (RDN) is achieved by the application of an isolated, low dose radiofrequency pulse to the renal artery endothelial surface via a percutaneous catheter-based procedure [71]. Most trials in humans, including HTN-3 and the SPYRAL HTN-OFF MED, have shown that renal denervation’s effect on BP has considerable variability [72,73]. 

However, the effect of this procedure in patients with resistant hypertension and OSA was not clear until 2011, when Witkowski et al. [74] studied 10 patients with refractory hypertension and OSA who underwent the procedure and then completed 3-month and 6-month evaluations. They observed decreases in both systolic and diastolic pressures at 6 months (−34/−13mmHg for SBP and DBP at 6 months, *p* < 0.01). Interestingly, there was also an improvement in sleep apnea severity. Following this, a phase II trial (testing a larger population to assess efficacy and side effects) was conducted in 60 patients with resistant hypertension along with coexisting moderate-to-severe OSA [75]. At 3 months, a decreased office and ambulatory BP along with a significant decreased in OSA severity was noted (AHI 39.4 in controls vs 31.2 in interventional group).

Thus, while repetitive apneic events result in markedly increased SNA, the responses seen following RDN suggest an important role of renal SNA in the development of resistant hypertension in OSA. Furthermore, RDN may not only lower BP, but also may attenuate apnea severity, in resistant hypertensive patients with sleep-disordered breathing. 

#### 5.2.3. Role of Beta blockers

Wolf et al. [76] hypothesized that beta blockers may diminish cardiac sympathetic overdrive caused by sleep disordered breathing in hypertensive patients with OSA. They demonstrated that patients on beta blockers had attenuated apnea-induced heart rate increases. They also observed that heart rate decelerations were similar in groups with and without beta-blockers. The incidence of ectopics and conduction abnormalities were comparable in both groups. 

## 6. Conclusions

Sympathetic neural responses to hypoxemia and hypercapnia result in marked nocturnal increases in adrenergic drive and BP. There is significant carryover of heightened sympathetic activation even during daytime wakefulness, in part due to tonic chemoreflex activation. Chemoreflex, baroreflex, and vasoactive mechanisms may contribute to an increased risk of hypertension in OSA (Figure S1).

However, both OSA and hypertension are common and hence may often exist as comorbidities. Not all patients with OSA and hypertension necessarily have hypertension occurring as a result of OSA. Hence, treatment of OSA may not always elicit marked decreases in BP.

OSA-related hypertension shows multiple neurogenic mechanisms of pathogenesis and these are potential mechanisms which have been targeted for the treatment of hypertension associated with OSA and treatment resistant hypertension. Neural modulation therapy, such as renal denervation, may have a role in lowering BP in OSA patients with resistant hypertension, in whom there is suggestive evidence that apnea severity may also be attenuated. Other neural modulatory strategies, such as hypoglossal nerve stimulation, are specifically targeted at reducing apnea severity, and may be able to lower BP on the basis of improved oxygenation and sleep quality in selected patients. Future studies should look to further evaluate the above approaches and identify other treatment strategies, including chemoreflex and baroreflex modulation, to attenuate apnea severity and/or neurogenic hypertensive consequences of OSA. 

## Supplementary Materials

The following are available online at https://www.mdpi.com/2077-0383/9/2/591/s1, Figure S1: Figure depicting various pathways leading to neurogenic hypertension.
