# Supplementary figures and images for: Sleep Apnea, Hypertension and the Sympathetic Nervous System in the Adult Population
Source: J Clin Med. 2020 Feb 21;9(2):591. doi: 10.3390/jcm9020591 (PMC7073618; doi:10.3390/jcm9020591)

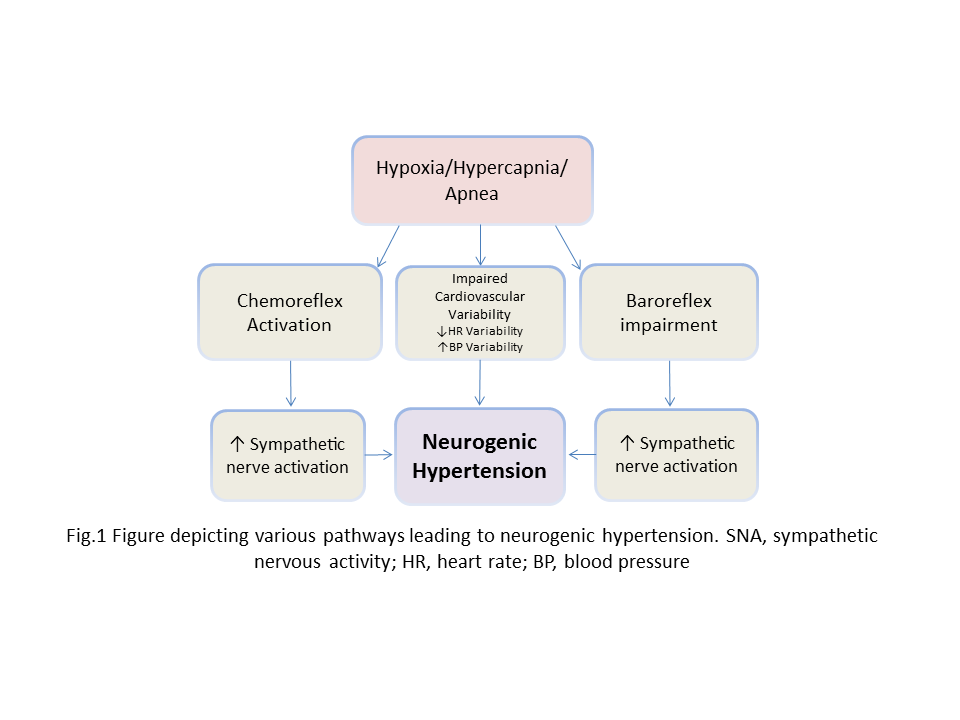

Supplement: Supplementary file 1 [file jcm-09-00591-s001.zip › Pathogenesis.tif]
